# Tractotomy using the Penrose drain guide for deep lung injury caused by chest drainage: a case report

**DOI:** 10.1186/s40792-021-01215-6

**Published:** 2021-06-01

**Authors:** Hironori Oyamatsu, Hideki Tsubouchi, Kunio Narita

**Affiliations:** 1 Department of Thoracic Surgery, Okazaki City Hospital, 3-1 Gosyoai, Koryuji-cho, Okazaki, Aichi 444-8553 Japan; 2 Department of Thoracic Surgery, Nagoya University, 65-Banchi, Tsurumai-cho, Showa-ku, Nagoya-shi, Aichi 466-8560 Japan; 3 Department of Thoracic Surgery, Toyohashi Municipal Hospital, 50 Aza Hachiken Nishi, Aotake-Cho, Toyohashi, Aichi 441-8570 Japan

**Keywords:** Penetrating lung injury, Pulmonary tractotomy, Penrose drain guide, Wound tract

## Abstract

**Background:**

Pulmonary tractotomy effectively treats deep pulmonary penetrating injuries; however, it requires the accurate insertion of forceps or a stapler into the wound tract. This report describes a case of tractotomy using the Penrose drain guide for a deep lung injury caused by chest drainage.

**Case presentation:**

A 75-year-old man suffered multiple rib fractures and hemothorax. After admission, chest tube drainage was performed because the patient’s respiratory condition deteriorated due to increased right pleural effusion. However, as the chest tube was stabbing into the right upper lobe, a pulmonary tractotomy was performed to treat the injury. Cutting the visceral pleura just over the tip of the chest tube caused the tube to completely penetrate the lung. A Penrose drain tube was fixed to the chest tube, which was then removed. The Penrose drain tube completely penetrated the lung and was coupled to the anvil side of the stapler to guide it smoothly into the wound tract. After stapling left the wound tract open, selective suture ligation of the damaged vessel and bronchioles was performed.

**Conclusions:**

Although the indications for tractotomy using the Penrose drain guide are limited, we believe that this technique can be useful in patients with deep stabbing or penetrating lung injuries with rod- or tube-shaped foreign body remnants.

## Background

Pulmonary tractotomy is effective for the treatment of deep pulmonary penetrating injuries; however, it requires the accurate insertion of forceps or a stapler into the wound tract. In this report, we describe a case of tractotomy using the Penrose drain guide for a deep lung injury caused by chest drainage.

## Case presentation

**Fig. 1:**
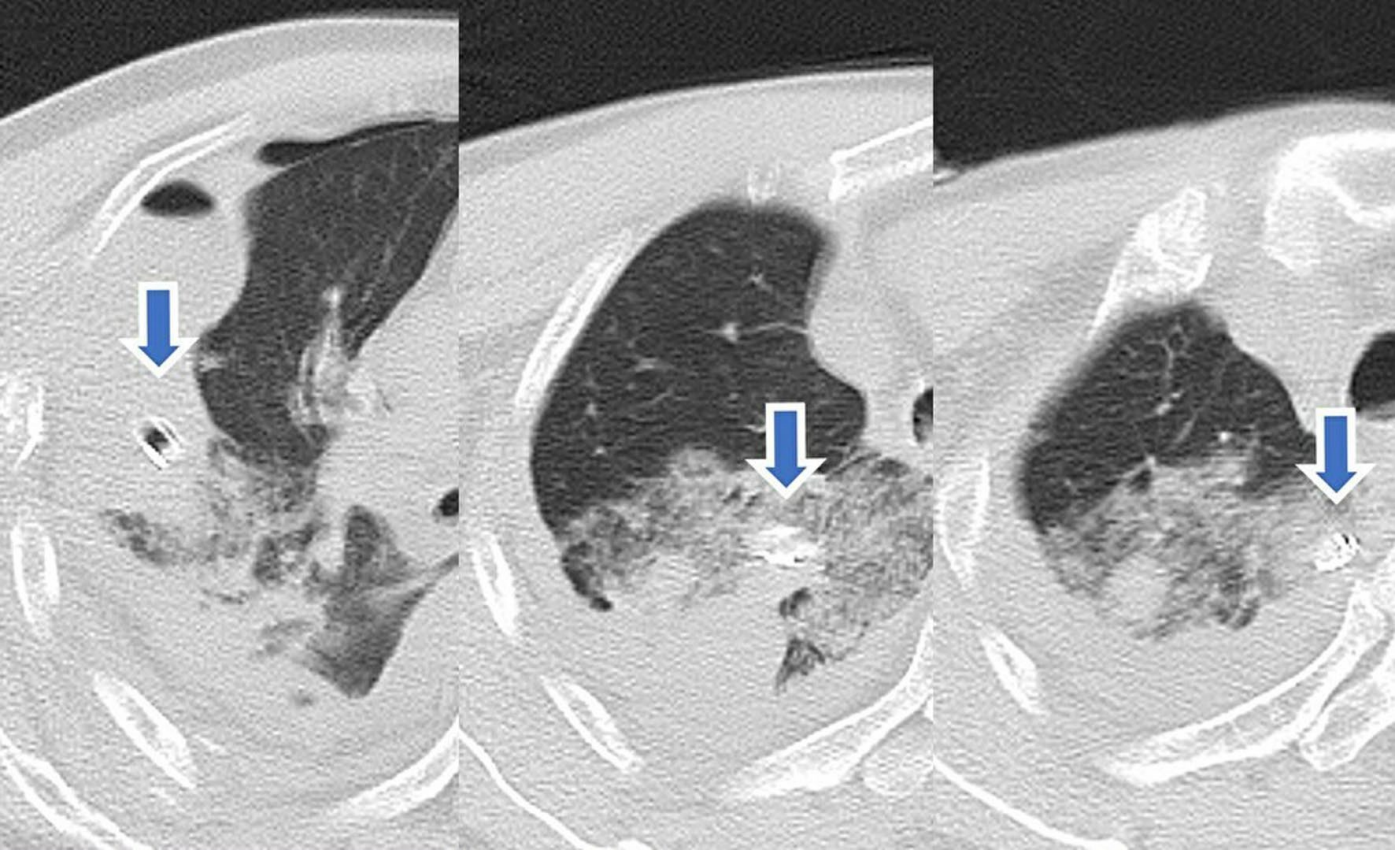
Computed tomography showing chest tube (arrows) stabbing the right upper lobe

A 75-year-old man with pulmonary emphysema slipped and fell from a cliff. He suffered multiple rib fractures and right hemothorax. After admission, the patient’s hemothorax and respiratory condition gradually worsened. On the 8th day of hospitalization, his respiratory condition deteriorated, and his breathing became labored due to increased right pleural effusion. Thoracic drainage was performed, and a chest tube (20 Fr) was inserted without resistance; however, the patient coughed up a large amount of blood, and computed tomography showed that the chest tube was protruding into the right upper lobe (Fig. 1). Emergency surgery was performed to treat the deep pulmonary stabbing injury.

**Fig. 2:**
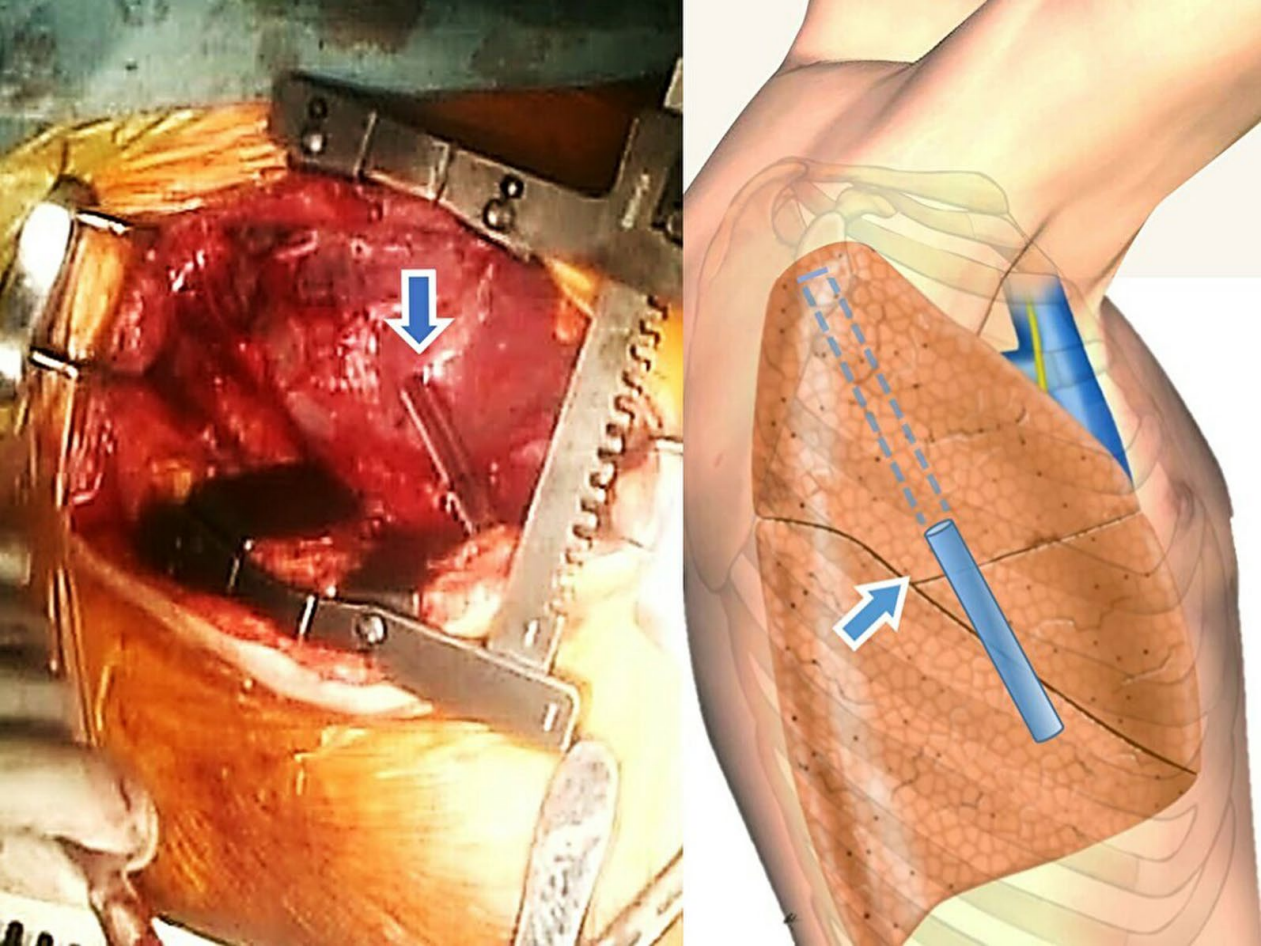
Operative findings showing chest tube (arrows) stabbing the right upper lobe toward the apical portion, which has not completely penetrated through the lung but has extended to just under the visceral pleura

**Fig. 3:**
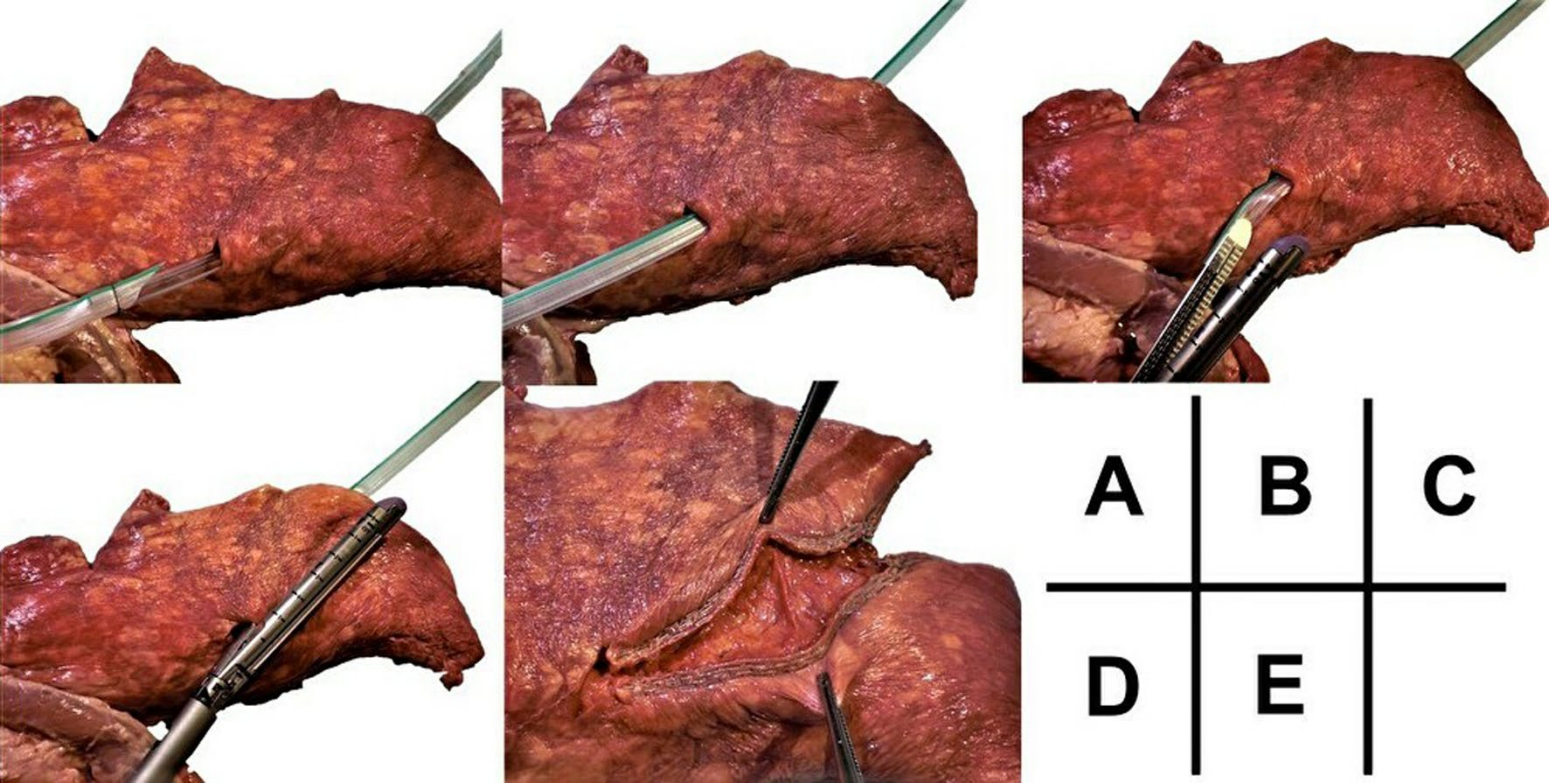
Procedure (pig lung models): **A** the Penrose drain is fixed to the chest tube, which is removed; **B** the Penrose drain is seen fully penetrating the lung. **C** The Penrose drain was attached to the stapler, **D** which was guided into the wound tract. **E** Stapling made the wound tract open

**Fig. 4:**
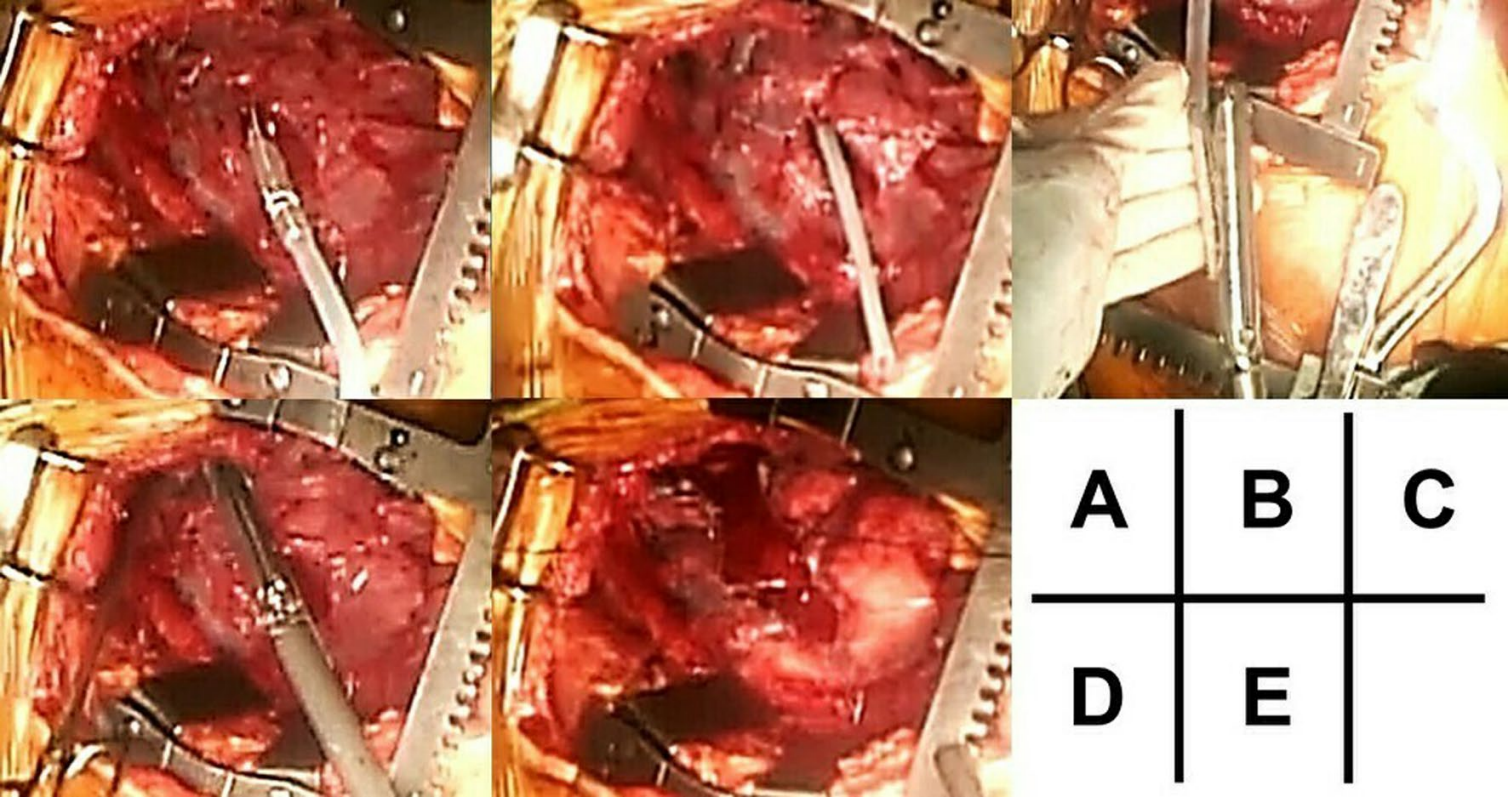
Procedure (surgical image): **A** the Penrose drain is fixed to the chest tube, which is removed; **B** the Penrose drain is seen fully penetrating the lung. **C** The Penrose drain was attached to the stapler, **D** which was guided into the wound tract. **E** Stapling made the wound tract open

The chest tube causing the pulmonary damage was retained in the wound tract to prevent air embolism and bleeding into the respiratory tract. The chest tube partially protruded into the right upper lobe toward the apical portion, just under the visceral pleura (Fig. 2). However, cutting the visceral pleura covering the chest tube’s tip caused the tube to fully penetrate the lung. A Penrose drain tube (outer diameter: Φ6.0 mm) was fixed to the chest tube, which was then removed. The Penrose drain tube fully penetrated the lung and was coupled to the anvil side of the stapler to guide the stapler smoothly into the wound tract (Figs. 3, 4). Stapling allowed the wound tract to remain open, and selective suture ligation of the damaged vessel and bronchioles was performed in the wound tract.

After the operation, the drain placed during the operation was removed on the 3rd day postoperatively. Although the ventilator's withdrawal was difficult due to sputum accumulation, it was successfully withdrawn on the 10th postoperative day. The patient was transferred to the rehabilitation hospital on the 19th postoperative day.

## Discussion

Wall et al. [1] reported the usefulness of tractotomy in deep penetrating lung injuries, while Asensio et al. [2] reported performing a tractotomy using a stapler. Using this technique for deep penetrating lung injury, thereby opening the wound tract, enables selective ligation of the vessels and bronchioles in the wound tract and preservation of the lung. This is a simple technique; hence, it has many advantages in terms of allowing direct control of bleeding and air leakage, reducing the operative time, as well as preservation of the lung. A lung-sparing technique, including tractotomy, is reportedly effective in 80% of patients with penetrating lung injuries, and tractotomy decreases morbidity and mortality [3]. In the present case, chest tube drainage was performed to treat respiratory distress due to hemothorax, and this procedure caused the pulmonary stabbing injury. The pulmonary parenchyma was considered vulnerable due to the presence of pulmonary emphysema; the drain was inserted without resistance. To control airway bleeding and repair bronchial injury, pulmonary tractotomy was performed promptly and successfully.

In this case, tractotomy using the Penrose guide technique with the stubbed chest tube was very effective. The described use of the Penrose drain tube guide is an easy and safe technique to introduce the stapler into the fused fissure during lobectomy [4]. Muraoka et al. (5) performed a tractotomy with gentle stapler insertion into a deep wound tract without fully penetrating the lung. However, adapting this to the vulnerable pulmonary parenchyma, such as in the present case, may cause further damage to the deep pulmonary parenchyma and vessels, with the stapler protruding out of the wound tract. Therefore, in this case, the stubbed chest tube that did not initially completely penetrate the lung was made to fully penetrate the lungs, and this penetration was used to pass the Penrose drain through the wound tract. Using the Penrose drain as a guide, the stapler was safely and accurately passed through the wound tract to perform tractotomy.

Certainly, the indications for tractotomy using the Penrose drain guide are limited. This technique requires a rod- or tube-shaped foreign body to be left in the lung so that the Penrose drain tube can be affixed to it. However, utilizing the Penrose guide facilitates the guiding of the stapler into the wound tract smoothly, avoiding damage to the wound tract from the stapler. It transforms the tractotomy into an easy, safe, and accurate procedure.

## Conclusions

Although the indications for tractotomy using the Penrose drain guide are limited, we believe that this technique can be useful in patients with deep stabbing or penetrating lung injuries with rod- or tube-shaped foreign body remnants.

## Data Availability

None.
